# Supplementary material for: Real-World Survival Impact of New Treatment Strategies for Lung Cancer: A 2000–2020 French Cohort
Source: Cancers (Basel). 2024 Aug 5;16(15):2768. doi: 10.3390/cancers16152768 (PMC11312246; doi:10.3390/cancers16152768)
Supplement: Supplementary file 1 [file cancers-16-02768-s001.zip › Supplementary Figure S1.pdf]

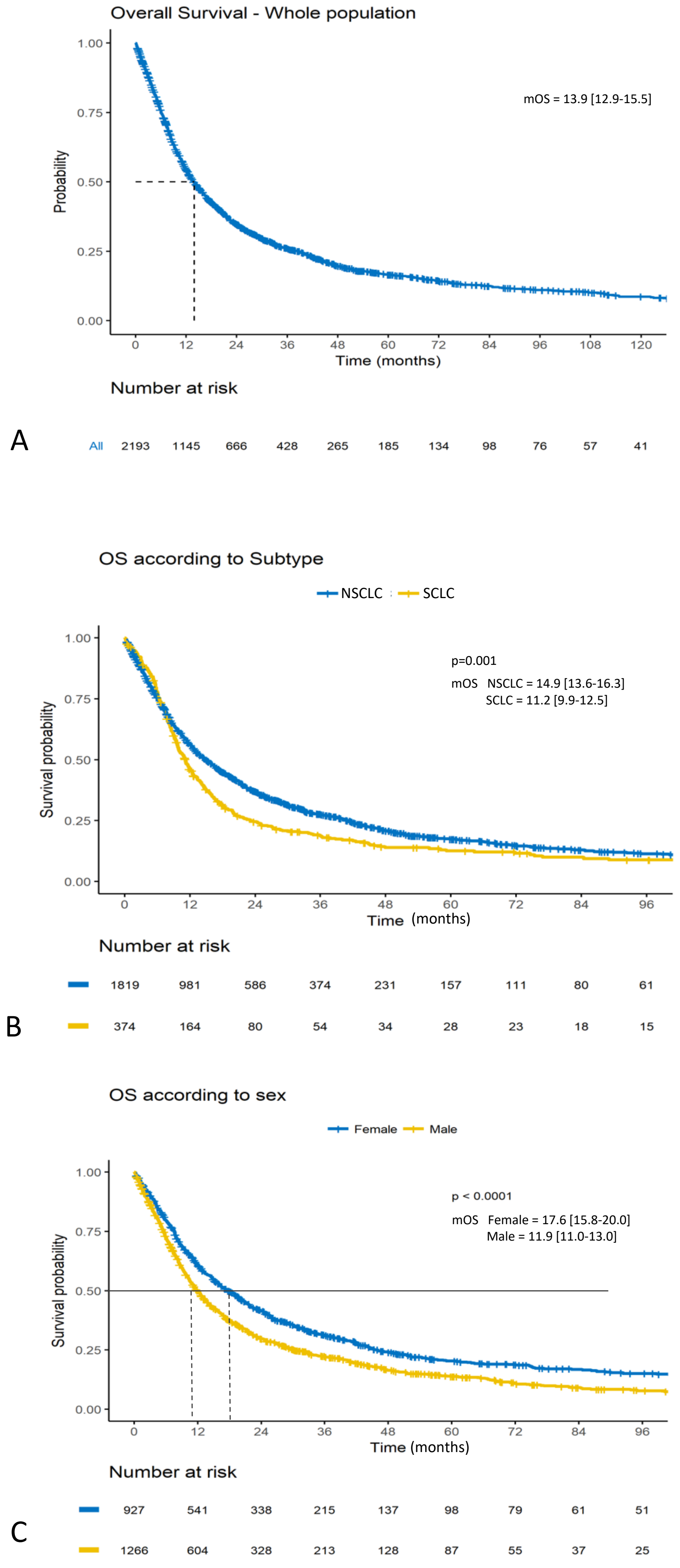

Supplemental Figure S1. Overall Survival curves in the whole cohort, accroding to histology and gender. (A) OS curve in the whole cohort. (B) OS curves according to histology. (C) OS curves according to sex. Legend : mOS = median overall survival (months); [IC95%]; NSCLC = Non Small Cell Lung Cancer; SCLC = Small Cell Lung Cancer.
