# Supplementary material for: Real-World Survival Impact of New Treatment Strategies for Lung Cancer: A 2000–2020 French Cohort
Source: Cancers (Basel). 2024 Aug 5;16(15):2768. doi: 10.3390/cancers16152768 (PMC11312246; doi:10.3390/cancers16152768)
Supplement: Supplementary file 1 [file cancers-16-02768-s001.zip › Supplementary Figure S2.pdf]

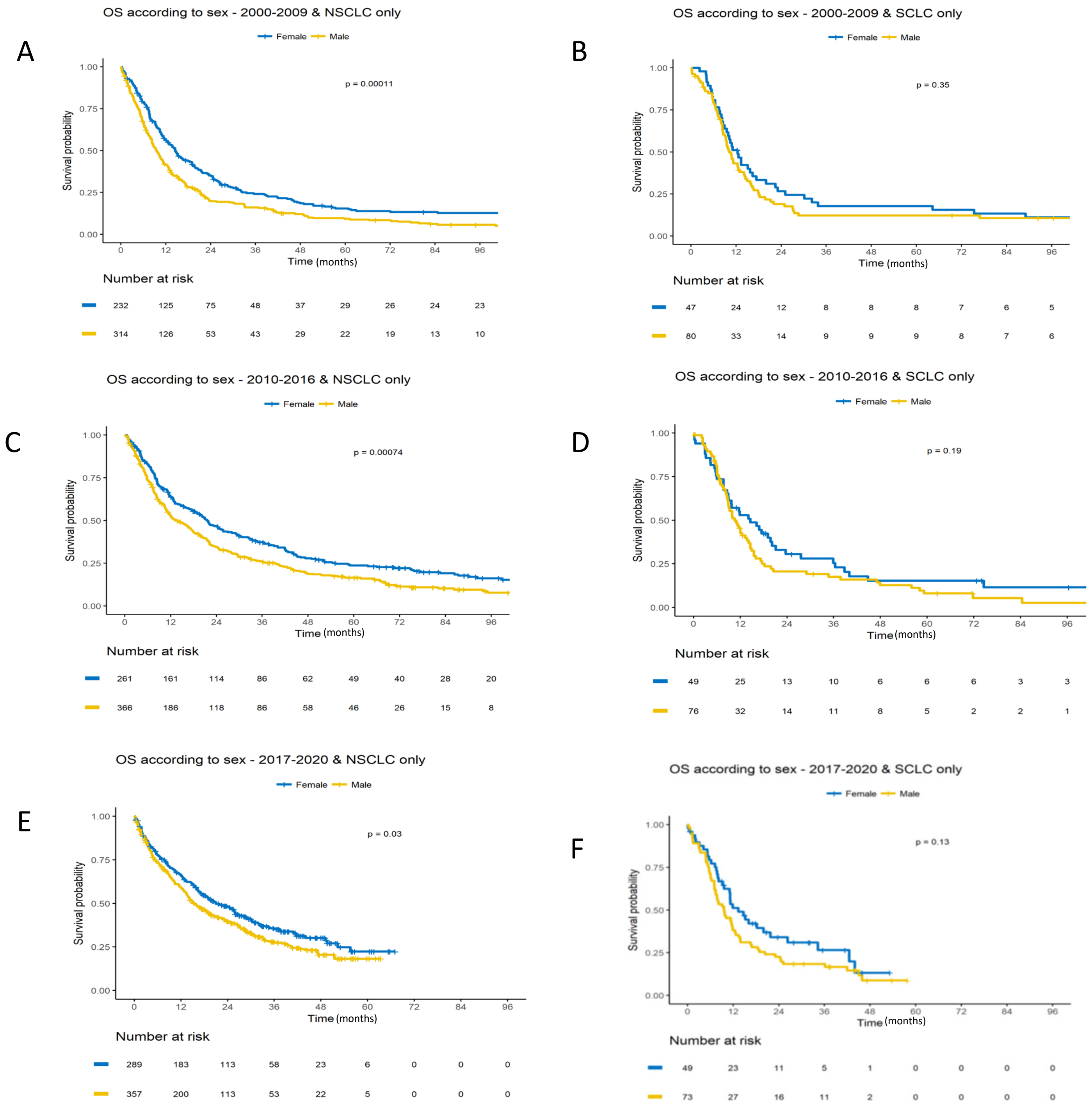

Supplemental Figure S2. Overall Survival curves according to gender and histology considering the 3 Periods. (A) NSCLC during Period-1. (B) SCLC during Period-1. (C) NSCLC during Period-2. (D) SCLC during Period-2. (E) NSCLC during Period-3. (F) SCLC during Period-3. Legend: NSCLC = Non Small Cell Lung Cancer; SCLC = Small Cell Lung Cancer.
